# Barriers for recess physical activity: a gender specific qualitative focus group exploration

**DOI:** 10.1186/1471-2458-14-639

**Published:** 2014-06-23

**Authors:** Charlotte Skau Pawlowski, Tine Tjørnhøj-Thomsen, Jasper Schipperijn, Jens Troelsen

**Affiliations:** 1 Research Unit for Active Living, Department of Sports Science and Clinical Biomechanics, University of Southern Denmark, Campusvej 55, 5230 Odense, M, Denmark; 2 Centre for Intervention Research in Health Promotion and Disease Prevention, National Institute of Public Health, University of Southern Denmark, Øster Farimagsgade 5a, 1353 Copenhagen, K, Denmark; 3 National Institute of Public Health, University of Southern Denmark, Øster Farimagsgade 5a, 2. 1353 Copenhagen, K, Denmark

**Keywords:** Focus groups, Physical activity, Children, Recess, Environmental barriers

## Abstract

**Background:**

Many children, in particular girls, do not reach the recommended amount of daily physical activity. School recess provides an opportunity for both boys and girls to be physically active, but barriers to recess physical activity are not well understood. This study explores gender differences in children’s perceptions of barriers to recess physical activity. Based on the socio-ecological model four types of environmental barriers were distinguished: natural, social, physical and organizational environment.

**Methods:**

Data were collected through 17 focus groups (at 17 different schools) with in total 111 children (53 boys) from fourth grade, with a mean age of 10.4 years. The focus groups included an open group discussion, go-along group interviews, and a gender segregated post-it note activity. A content analysis of the post-it notes was used to rank the children’s perceived barriers. This was verified by a thematic analysis of transcripts from the open discussions and go-along interviews.

**Results:**

The most frequently identified barriers for both boys and girls were weather, conflicts, lack of space, lack of play facilities and a newly-found barrier, use of electronic devices. While boys and girls identified the same barriers, there were both inter- and intra-gender differences in the perception of these barriers. Weather was a barrier for all children, apart from the most active boys. Conflicts were perceived as a barrier particularly by those boys who played ballgames. Girls said they would like to have more secluded areas added to the school playground, even in large schoolyards where lack of space was not a barrier. This aligned with girls’ requests for more “hanging-out” facilities, whereas boys primarily wanted activity promoting facilities.

**Conclusion:**

Based on the results from this study, we recommend promoting recess physical activity through a combination of actions, addressing barriers within the natural, social, physical and organizational environment.

## Background

Like in many other countries, a large number of Danish school children do not reach the recommended minimum level of 60 minutes of moderate-to-vigorous physical activity (MVPA) per day [1] and physical activity (PA) decreases significantly between ages 9 and 15 years [2]. Engaging in PA has positive effects on both the physical and mental health of children [3-6]. Children spend a substantial amount of their waking hours at school and since recess PA can contribute with up to 40% of children’s recommended daily PA, school recess provides many opportunities for children to be physically active [7]. Targeting school recess periods is important from a health perspective [8] and school-based PA, especially recess PA, has been shown to improve cognitive performance, academic achievement, classroom behavior, attention and concentration [9].

Evidence shows that, in general, boys are more active than girls [10], also during recess [7,11,12]. One study in particular reported that the greatest gender difference in children’s PA was found in institutional settings, such as schools, where children relied on self-organized activities during recess and after-school day care [13]. Identifying factors affecting children’s recess PA, with a focus on gender differences, will aid in informing school policy and developing strategies designed to promote PA in school settings.

To date, research on recess PA has predominantly focused on quantitative measures of correlates of PA, using cross-sectional surveys and school-based intervention studies [11]. The quantitative surveys typically focused on a narrow set of predefined factors, often constructed by adults [14]. To really understand the factors affecting PA it is crucial to listen to children and understand their perspectives [14].

Two comprehensive Australian studies have explored children’s barriers for recess PA from a qualitative perspective and identified a lack of facilities/equipment, bullying, school policy, clothes, lack of teacher support [15,16], lack of space, weather [15], playground aesthetics, fundamental movement skills and recess duration [16] are important barriers to recess PA. However, these two studies did not take gender perspectives into account, and little is known about gender differences in PA during recess [11].

There is increasing evidence that the environments we live in have an impact on our behavior, including our inclination to engage in PA [17,18]. The current study builds on a comprehensive socio-ecological model positing that PA behavior results from multiple influences [19]. Inspired by this socio-ecological framework, four groups of barriers have been identifıed: natural environment (e.g. weather, topography and air quality), social environment (e.g. interpersonal relations and social climate), physical environment (e.g. facilities and surroundings) and organizational environment (e.g. policy and rules).

The aim of this study was to explore gender differences in children’s perceptions of barriers to recess PA by using a qualitative approach and the socio-ecological model as a theoretical framework.

## Methods

The study is the first phase of a larger Danish schoolyard intervention study: The Activating Schoolyards Study, aiming to improve children’s opportunities to become physically active in the schoolyard during recess, particularly the least physically active schoolchildren. All schools in Denmark were invited to participate in the study. Out of the 106 schools that submitted a participation proposal 17 were selected by an expert panel and included in this study. The results from this study will be used as one of the inputs to the planning process of interventions at selected schools in the next phase of The Activating Schoolyards Study. The planning of the specific interventions is driven by the local actors at each school.

The 17 schools represent a wide range of schools. As shown in Table 1, the 17 schools varied in geographic location, school type, number of pupils and their grade-levels, socioeconomic status (based on parental income), square meters of schoolyard per child, recess rules and number of play facilities. All but one school allowed the use of electronic devices during recess.

**Table 1:** Main characteristic of the 17 schools in the study

**Characteristics**	**Number of schools**
	**n = 17 (100%)**
Region:	
Capital region	4 (24)
Region Zealand	2 (12)
Region North	2 (12)
Central Denmark	5 (28)
Southern Denmark	4 (24)
School type:	
City	12 (71)
Urban	5 (29)
Number of pupils:	
> 600	9 (52)
400-600	4 (24)
< 400	4 (24)
Number of fourth grade classes per school:	
1	3 (18)
2	5 (29)
3	7 (41)
4	2 (12)
Fourth grade pupils’ relative hierarchical position at the school location:	
The oldest	4 (24)
The “in-betweens”	12 (70)
The youngest	1 (6)
Parents income range*:	
≥ average school in Denmark	8 (47)
< average school in Denmark	6 (35)
Size of schoolyard per child (m^2^):	
< 10	4 (24)
10-49	7 (40)
50-99	4 (24)
> 100	2 (12)
Recess rules for fourth grade:	
Must stay out in recess	4 (24)
Must stay out in recess during summertime	3 (18)
Must stay out in one of the two main recesses	5 (29)
The children must decide themselves	5 (29)
Number of play facilities for fourth grade:	
<10	3 (18)
10-15	5 (29)
16-20	8 (47)
>20	1 (6)
Use of electronic devices allowed in recess:	
Yes	16 (94)
No	1 (6)

*Published data from Statistics Denmark. Three schools are not included why they have been merged after the calculation.

In Denmark, school is mandatory for children aged 6–16. Public schools are free of charge and children do not wear school uniforms. Schools are typically organized in three tiers: junior (grade 0–3, 6–9 years old), middle (grade 4–6, 10–12 years old) and senior (grade 7–10, 13–16 years old). Each class has a maximum of 28 gender-mixed pupils [20]. In fourth grade, children attend school for 24.5 hours a week, of which 135 minutes are allocated to physical education (PE) [21]. Approximately 60 minutes are dedicated to recess per day, being distributed over two to four breaks. In general, the lunch recess is the longest break, lasting 25–30 minutes.

The study adheres to the RATS guidelines for reporting qualitative studies. It was approved by the Danish Data Protection Agency (DOK230123). In addition, the study was approved by the school principals and informed consent was obtained from the parents of the focus group participants. The schools and children included in the study were anonymized by giving the schools numbers and changing the children’s names.

### Research design and procedure

To obtain an in-depth insight into children’s perceptions of barriers to recess PA, a qualitative research design was used. Focus groups were selected for this study as the most suitable technique as they have been proven to be an effective method in gathering data among children because they create interactive conversation, evoke memories, help participants to verbalize their responses and enable testing the consistency of statements [14,22,23].

Data were collected during a one-day visit to each of the 17 schools between April and June 2013. The school principal, or a designated teacher, was asked to identify three boys and three girls from the various fourth grade classes (10–11 years), who would represent differing levels of PA. Fourth grade pupils were selected to get an understanding of barriers to PA amongst this age group particularly in the context of the decline in PA from childhood to adolescence [2]. Seventeen focus groups (one at each school) were conducted. In total 111 children (53 boys and 58 girls), with a mean age of 10.4 years, participated in the focus groups. The group-size ranged from five to ten participants.

The focus groups lasted for approximately 60 minutes and were conducted during school hours. The focus groups were filmed using an iPad mini to record interactions [24] and to document who said what. To ensure consistency all focus groups were conducted and transcribed by the lead author.

Using the socio-ecological model as a theoretical framework, a number of questions were developed to prompt information about natural, social, physical and organizational environmental influences, as outlined in Table 2. The procedure and questions were pilot tested at two of the 17 schools.

**Table 2:** Procedure and questions used for the focus groups

**Phase**	**Activity**	**Setting**	**Duration**
1. Open focus group discussion	Firstly, informal conversation and *ice-breaking* activities were used to create a relaxing environment. Then a discussion was conducted to identify barriers that influence children’s recess play. Questions used were:	Classroom/meeting room at the school	30 minutes
	• What are your experiences around recess?		
	• What do you do during recess?		
	• Who are you playing with?		
	• Who initiates play?		
	• Is there something you want to do in recess that you cannot or may not do? What was this?		
	• What do you think about your schoolyard?		
	• Can you explain what physical activity is?		
	• Do you like being physically active? Why/why not?		
	• What influences your physical activity during recess?		
2. Go-along interview	The children pointed out where they usually played during recess, the activity they played and who they played with. A Google Earth aeria photograph of the schoolyard was used as a tool (by the moderator), to indicate where different activities took place.	Shared indoor and outdoor areas at the school	20 minutes
3. Post-it note activity	An open brainstorm to identify significant barriers to engaging in physical activity during recess. The groups were told to write down all the barriers that they could think of on post-it notes.	Classroom/meeting room. Gender separated groups	10 minutes

To facilitate the discussion and evoke memories, each focus group included a go-along group interview in the schoolyard, where the children showed the moderator places or spaces in the schoolyard that they used during recess [25]. An A3 sized Google Earth aerial photograph of the schoolyard was used as a visual tool [15,26]. Symbols, representing various activities, were placed on the map by the moderator to indicate where different types of activity took place.

At the end of the focus group discussions an open brainstorm session was conducted. The groups were told to write down all barriers that they could think of on post-it notes. In contrast to the rest of the focus group activities, the post-it note activity was done in gender segregated groups. This was to be able to study both inter- and intra-gender differences in the perceptions of barriers to recess PA.

Using multiple qualitative methods in the focus groups allowed for triangulation of the results as the different methods supplemented each other and provided a more complete picture [24,27].

### Data analysis

Recordings from the focus groups were transcribed after each focus group and the analytical process began during data collection whereby initial insights were used to refine the guide used for structuring the focus groups [28]. Upon completion of data collection, focus group transcripts and post-it notes were ordered with the explicit purpose of identifying barriers influencing engagement in recess PA across the schools [29]. The importance of barriers was deducted from those listed most frequent on post-it notes, those that took up much time during the interview, or were discussed with a lot of enthusiasm by the children.

As a first step, an overview of the range of barriers identified in the 17 focus groups was created to guide the development of a set of barriers perceived by the children [30,31]. Data from the post-it notes were analyzed using a deductive content analysis process involving coding, categorization, and summarizing [32]. The data were coded and arranged under headings derived from the social-ecological model (i.e. natural, social, physical and organizational barriers). Under each heading the coded comments were clustered into categories based on similar content. Then a thematic analysis was conducted to produce an in-depth description and understanding of the transcripts from the focus groups’ open discussion and go-along interview [30,31]. Phrases from transcripts that referred to barriers were highlighted and grouped, from which themes and subthemes emerged. Since themes were established based on a triangulation of different data sources, this process adds to the reliability of the study [33].

Finally, the data were examined from a gender perspective focusing on similarities and differences between boys’ and girls’ participation, activities and expressions in relation to recess PA.

## Results

Based on the post-it note activity 16 different barriers were identified: one natural barrier, four social barriers, seven physical barriers and four organizational barriers. Each of these barriers had varying degrees of perceived importance (Table 3).

**Table 3:** Perceived barriers to recess PA mentioned in the post-it note activity

**Barriers mentioned in post-it note activity**	**Total %**	**Girls %**	**Boys %**
	**(n = 34)**	**(n = 17)**	**(n = 17)**
*Natural*			
Weather	50	65	35
*Social*			
No-one to play with/not allowed in group play	9	12	6
Conflicts (disagreement, dominance)	41	35	47
Peer influence	15	18	12
Lack of teacher support/delayed by teacher	9	12	6
*Physical*			
Occupied play facilities	12	12	12
Lack of maintenance	15	12	18
Lack of grass areas	9	12	6
Lack of space	29	29	29
Lack of play facilities	68	71	65
Lack of access to play facilities	12	12	12
Boring play facilities	6	6	6
*Organizational*			
Recess duration	12	12	12
PE prior to recess	6	6	6
Allowed to stay inside	6	6	6
Use of electronic devices	29	29	29

For both boys and girls the five perceived barriers mentioned most were: weather, conflicts, lack of space, lack of play facilities, and use of electronic devices. These five barriers were also prominent in the focus groups’ open discussion and go-along interview. The post-it note activity showed no gender differences for these five barriers. However, the open discussion and go-along interview data showed that boys and girls perceived these five barriers differently. The following sections provide an in-depth description of these five barriers from a gender perspective.

### Weather – a natural barrier

Bad weather conditions seemed to be one of the main barriers to recess PA. Many children did not think it was fun to play outside in rainy or snowy weather. Some children commented that snow and rain stopped them from using facilities such as courts and fields for ballgame. Others said that they felt “freezing” and that bad weather conditions did not motivate them to participate in outdoor activities. Girls especially expressed bad weather conditions as a barrier to recess PA. One girl said:

“It’s not about bad weather but about how you feel about the weather […]. We like it when it’s a bit warm because then it’s much more fun to be outside because you can do more. When it’s cold or rainy then you really don’t want to do so much”. (Girl, school 12)

While girls preferred to stay indoors during recess doing sedentary activities when the weather was cold or rainy this was not the case for all boys, with some preferring to be in the playground regardless of weather conditions. One boy said:

“I am definitely outside playing even if it is raining”. (Boy, school 12)

While weather conditions are uncontrollable it was interesting to note that the effects of weather conditions seemed to be strengthened by the school’s recess policy. At many schools the children could decide for themselves whether they wanted to stay inside during recess doing sedentary activities, particularly in the winter and autumn season (Table 1). Additionally at many schools the children were not allowed to use some outdoor areas during rainy weather in order to prevent dirt being carried indoors. Three schools allowed children access to the sports hall during recess, which both boys and girls liked.

### Conflicts - a social barrier

At almost every school social relations during recess were a topic of great discussion. The majority of children identified conflicts, caused by disagreement and dominance, as an element that disrupted play. Both boys and girls often argued about what to play, where to play and who was allowed to participate in the play. Boys in particular had conflicts when playing soccer or other ballgames. The reason for such conflicts was often caused by the importance placed on winning. Many of the boys took the ballgame so seriously that team constitution and rules of play often caused disagreement and sometimes even fights. A conversation between the moderator and three boys highlighted this:

Moderator: Are there often conflicts?

Michael: Yes, at the soccer field

Ben: Almost every day

Nick: Often somebody fights, but not every day

Moderator: What are they fighting about?

Ben: If it is a goal or a free kick

Nick Or hand ball. They were fighting today about if there was a hand ball

Ben: Alex, he just wants to win (school 2)

Conflicts caused by dominance were also experienced as a significant barrier to recess play. Boys felt dominated by older boys who “wrecked” their play by taking their equipment (e.g., balls), facilities (e.g., soccer field) or disrupting games (e.g., throwing snowballs). This both ruined their play and started conflicts which they felt were time consuming and a waste of time. One boy said:

“The older ones can just, well, be a little annoying when they come and say that they had the soccer field first. Then we have to find a teacher and it ends up that we have to leave because they are lying. They just say: “We had it first”. It is quite annoying”. (Boy, school 17)

Many girls wanted to play ballgames but they realized that the ballgame areas were dominated by boys. Girls felt that they were not allowed to join the boys’ ballgames or if they were allowed to join, that boys did not include them in the game (e.g. boys did not pass the ball to them), meaning the girls stood passively waiting for the ball. At a few schools, where there were several soccer fields, girls were playing soccer by themselves, however, at most schools the only opportunity for girls to play soccer was by joining the boys. One boy and two girls discussed this as follows:

Rita: They do not want girls to take part [in soccer games] because they are not good enough

Simon: In my class girls are allowed to take part

Isabella: But then they do not pass the ball to us

Simon: Yes exactly, I think that the boys say yes to them so that the girls do not complain to the teachers (school 15)

The children realized it was difficult to solve the conflicts by themselves and that it took a lot of effort and time during recess. They did not think the teachers were of any help because it was difficult to find a teacher in the schoolyard. One boy explained:

“You actually use all your recess finding a teacher first and then you can throw them off [Older pupils from the soccer field]”*.* (Boy, school 7)

Some, mainly girls, thought it would reduce conflicts and create more play across the genders and different age groups if teachers were involved as play initiators, creating teams or acting as referees.

### Lack of space – a physical barrier

The number of square meters per child for fourth grade children differed widely between schools (Table 1). Children reported feeling “crowded” in the schoolyard at schools with small outdoor areas and lots of children. It complicated recess PA as many children were doing different activities in the same area at the same time and often they bumped into each other which led to conflicts. One girl said:

“We went to another school before where there was really a lot of space and we never started arguing about anything because there was so much space”. (Girl, school 16)

Because of overcrowding and excessive noise in the small schoolyards it was mentioned that in particular girls, often sought out small secluded areas where they could stay in smaller groups. Even though children at some schools were not allowed to stay indoors during recess, indoor areas were popular places for these girls to go to for quiet sedentary activities. A conversation between the moderator and three girls highlighted this:

Lana: Typically, we sit on those couches and just talk [At the library]

Alba: In fact, we are not allowed to stay in here at all but we [girls] need to have a place to stay

Moderator: So you wish that you were allowed to stay here?

Catharina: Yes because there are not so many [children] in here so it’s quiet (school 7)

The fact that girls expressed a lack of space seemed not only to be related to having a small schoolyard, but also to the desire of having smaller areas for themselves. Even at schools with plenty of space per child, many girls were still attracted to smaller secluded areas.

At one school with a small schoolyard the school allowed the oldest pupils to go to a nearby park during recess. At this school it was attractive to go to the park as the older pupils had it all to themselves and therefore they took advantage of their special privilege. One girl expressed it as:

“It’s nice that you just can go there [to the park] and say “whew” now there are not so many [children]”. (Girl, school 14)

### Lack of play facilities – a physical barrier

While the number of play facilities at each of the schools varied widely, the principal barrier identified to recess PA was a lack of schoolyard facilities, defined as both buildings (e.g. gymnasiums), courts or equipment (fixed/unfixed) (Table 1). Almost every child mentioned facilities they did not have, or had but which did not live up to their expectations. At those schools where children were allowed to stay indoors (under certain circumstances) (Table 1), many children preferred to stay in the classroom during recess because of the perceived lack of play facilities. One boy stated:

“I mostly like to stay indoors. I do not really think there is anything to do outdoors […]. Well, we do not have any grassy soccer field. I miss that and some larger goals”. (Boy, school 7)

Even though all schools had soccer fields of some sort, the most wanted play facilities among the children, in particular boys, were soccer related. Many boys expressed a need to be physically active during recess and they wanted facilities they could use for PA (e.g. climbing facilities for tag, a large slide, an obstacle course, or skateboard and parkour facilities). Girls also wanted climbing facilities, but with another use in mind. Many girls requested facilities which could provide a smaller cozy place where they could hang out and isolate themselves in small groups. Bird’s nest swings and small huts were other examples of sought-after facilities among girls. A dialogue between the moderator and three children went as follows:

Henrietta: It could be nice with swings, for instance such big swings like a nest because they are cozy and then you can sit down there and talk and you still get fresh air and at the same time you have fun

Maria: Yes, and climbing nets so you can climb up and down and then there is a little hut up there where you can sit and talk

Moderator: Boys, do you also need swings?

William: Not that much

Henrietta Maybe it’s not for soccer boys but it’s cozy for girls (School 1)

Most play facilities that were provided were quickly occupied and the lack of facilities also resulted in a rush to get to the facilities first. The children pointed out that in particular, the soccer fields and swings were often occupied. This meant they had to eat their packed lunch quickly and in some cases ask their teacher if they could begin recess earlier to get to the facilities first. If the boys did not get the facilities they wanted, they were often very creative in playing something else or using alternative facilities (e.g. benches were used as soccer goals, door sills and stairs as ramps for skateboards and scooters and playhouse roofs as parkour facility). In contrast, girls engaged in more passive activities when the facilities they wanted to use were occupied. Two girls explained what they did when facilities they wanted were occupied:

“Then we have to stay next to the swings and wait until they leave”. (Girl, school 12) “Then we just go into our classroom and talk”. (Girl, school 8)

### Use of electronic devices – an organizational barrier

At the 16 schools which allowed the use of electronic devices during recess (Table 1), almost every child in the fourth grade brought a smartphone or tablet to school on a daily basis. In addition to that, children at five of the schools were allowed to use library or classroom computers during recess. Both boys and girls used computers, smartphones and tablets for gaming, Facebook, YouTube, Instagram and to play music. Many of the children stated that their smartphone or tablet was tempting to use during recess periods. One boy commented:

“It attracts us like a magnet”. (Boy, school 9)

However, they also reported that allowing the use of those electronic devices during recess acted as a barrier to getting fresh air, socializing, improving their concentration and PA. Use of electronic devices was mostly perceived as a barrier for recess PA by those who preferred to play physically active games. Some children pointed out that there were not enough children for group play because many of their classmates were absorbed in their mobile phone. Furthermore, some children reported playing on a computer or smartphone during recess because of peer pressure even though they would rather do something physically active. A conversation between the moderator and four children went as follows: 

Simon: Sometimes I think it is a bit annoying that everybody sits at the computers […]. I’m actually the only one who runs around outside while everybody else sits and plays on the computer

Moderator: Why is it annoying?

Harry: Because we are not really together and it is very boring

Sally: I think it is a bad rule

Harry: Yes, I think they [the school management] should make a new rule so you were only allowed to stay inside playing on the computer in the lunch break and it was closed down in the other recess periods

Sally: If they were not allowed to sit at the computers or in the classroom then everybody would be outside, you see

[…]

Moderator: What would you [addressed to Tom, who plays on the computer every recess periods] do if you were not allowed to play on the computer?

Tom: Then I would play soccer outside

Moderator: If you could choose between playing on the computer or soccer what would you choose then?

Tom: Soccer

Moderator: But why then are you playing on the computer?

Tom: Just because my friends do (school 15)

As mentioned in the above conversation, many children thought that using computers or smartphones during recess was getting out of control because of the barriers it caused, and thought it necessary to reduce the use of electronic devices. Some thought rules were needed and having “screen breaks” on some of the school days or in some recess periods were mentioned as a solution. Others suggested that more play facilities in the schoolyard could solve the problem.

## Discussion

The present study set out to contribute to the current literature about children’s recess PA by examining and describing children’s perceptions of barriers to school recess PA, including identification of why recess PA differs between boys and girls. Five key barriers to recess PA emerged: weather, conflicts, lack of space, lack of play facilities, and use of electronic devices. Boys and girls identified the same barriers as the most important, but dealt with the barriers differently.

Cold and rainy weather conditions were identified as a significant barrier to recess PA. This was in contrast to Ridgers et al. who found no significant variation in children’s level of recess PA across varying daily weather conditions or seasons [34]. This variation could be due to climatic differences between the studies (UK versus Denmark). Another explanation could be that the children in the study of Ridgers et al. had no option to play inside, whereas the majority of the current study’s schools let the children stay indoors during winter and bad weather, which supported more sedentary activities and especially those activities that girls choose to do. The importance of the school’s policy on recess PA was also seen in an Australian qualitative study where children had to stay indoors in both wet and hot weather conditions [15].

In the present study, conflicts were perceived as time consuming and a barrier to recess PA, especially among competitive sports-minded boys. Another study also found that conflicts were time consuming in PE lessons, suggesting that up to one quarter of lesson time was taken up by conflicts related to organization of teams, activities and game rules [35]. The lack of teacher present in outdoor areas seems to be related to conflicts, hence increased teacher supervision could lead to faster conflict resolution and thus provide increased PA, particularly among boys [36,37]. However, in our study girls also described benefiting from increased teacher supervision, in particular if the monitoring teachers participated in the play then girls experienced reduced conflicts and less boy dominance.

Lack of space was perceived as an important barrier to recess PA, which was similar to other qualitative studies [15,38]. This is also supported by findings from quantitative studies where more play space per child was positively associated with recess PA [39,40]. Conversely however, Sallis et al. found that play area size was not significantly associated with recess PA [37]. However, their study assessed available space in different schoolyards rather than the space available per child. In our study both boys and girls felt that lack of space was a barrier, but girls also verbalized a desire for smaller secluded areas, possibly because boys tend to dominate the main areas of the schoolyard [41-43]. Some studies have suggested that recess strategies to increase PA should consider reducing the dominance of soccer in schoolyards by allocating specific areas for other activities and thereby provide more space for those who do not want to play soccer [44,45]. However, in our study many girls also indicated they wanted to play soccer.

The most commonly mentioned barrier to recess PA, perceived by both boys and girls, was a lack of play facilities. This is in line with previous qualitative studies [15,16,46]. A review also found a strong positive association between recess PA and overall facility provision as well as the provision of unfixed equipment [11]. Similarly Zask et al. reported that the ratio of balls to children was related to vigorous physical activity (VPA) [47]. In this study both boys and girls emphasized a lack of soccer facilities and equipment, but there were gender differences in the most desired facilities. Boys primarily preferred physically activity promoting facilities (e.g., multi courts, obstacle course, climbing frames, skateboard and parkour facilities) whereas girls tended to prefer smaller secluded places where they could hang out and isolate themselves in smaller groups (e.g., bird’s nest swings, climbing frames and small huts).

In addition to those barriers which have previously been identified [11], this study also found that the use of electronic devices during recess were seen as a barrier to PA. While the children felt attracted to using electronic devices during recess, they also realized the use of electronic devices had consequences in relation to PA. A study, in a non-school setting, found that the young people with higher levels of computer use were the most inactive and were more likely to report computer use as a barrier to PA [48]. Conversely, children’s use of mobile phones when playing away from home has been found to be a facilitator for play because it helped alleviate parents’ safety fears [49]. We found that the use of electronic devices was not only a barrier for recess PA among the children who used electronic devices, but also for their classmates who preferred to play group games. The use of electronic devices seemed so widespread that the children themselves thought it necessary to reduce use of electronic devices, suggesting restrictions or more play facilities in the schoolyard. The reason electronic devices have not been previously identified as a barrier to recess PA is probably due to electronic devices being a relatively new phenomenon. Additional research is needed to explore the impact of this new barrier to recess PA and suggestions for future directions with regard to this finding are needed.

### Strength and limitations

A strength of this study is the use of multiple methods and analysis strategies. This facilitated in attaining a much richer form of data and greater credibility of results. Using this method at 17 relatively different schools involving 58 girls and 53 boys strengthens the transferability of the study. The consistency of findings from the children across the 17 different schools underpins that barriers identified are prevalent throughout a variety of school environments and widely recognized by children.

A limitation of the study was that there was only one focus group at each of the 17 schools. More focus groups at each school would have enabled a more detailed description of the schools. However, the 17 schools are as institutions homogeneous in structure and our purpose was not to create deep descriptions of each school. We preferred to look across the different schools included in the Activating Schoolyard Study [29]. Another limitation is that the focus groups only included fourth grade children. Perspectives of adolescents, teachers, school management and parents may differ from the children’s viewpoints. However, it was a deliberate choice only to study children’s perceived barriers. This decision was made in line with the new paradigm of childhood which states that children’s culture is worthy of study in its own right, independent of the perspective and concerns of adults [50]. Moreover, our findings indicate that the children are keenly aware of the importance of the barriers to recess PA. Throughout the focus groups, the children clearly articulated how their perceived barriers created significant obstacles to establishing healthy behaviors during recess and they suggested ways to mitigate some of these barriers.

## Conclusion

Five key barriers were identified by both boys and girls: weather, conflicts, lack of space, lack of play facilities, and a newly-found barrier, use of electronic devices. While boys and girls identified the same barriers, there were both inter- and intra-gender differences in the children’s perceptions of these barriers. These findings suggest that there is a need to use this methodology to better understand the barriers from a gender perspective and to search for new barriers in order to provide a more complete description of influences on children’s PA behavior during recess.

We recommend that school recess PA is promoted through a combination of actions that address barriers within the natural, social, physical and organizational environment. This implies using a socio-ecological approach focusing on different settings, e.g. implementing school policies which supporting activity in all weather conditions, more teacher presence during recess, recess activities organized by older students or teachers, creation of outdoor boy and girl zones, organization of student-driven play equipment stations, and regulations of electronic devices, particularly smart phones and tablets, during recess. These recommended actions are relatively low-cost, but require a high degree of commitment and motivation from both school management and teachers to be successfully implemented.

## Abbreviations

MVPA: Moderate to vigorous physical activity; PA: Physical activity; PE: Physical education; VPA: Vigorous physical activity.

## Competing interests

The authors declare that they have no competing interests.

## Authors’ contributions

CSP carried out the focus groups, analyzed the data and drafted the manuscript. TTT and JT participated in analyzing the data. JS participated in drafting the manuscript and analyzing the data. CSP and TTT designed the study. CSP and JT conceived the study. All authors read and approved the final manuscript.

## Pre-publication history

The pre-publication history for this paper can be accessed here:

http://www.biomedcentral.com/1471-2458/14/639/prepub
